# Chronobiomics: The Biological Clock as a New Principle in Host–Microbial Interactions

**DOI:** 10.1371/journal.ppat.1005113

**Published:** 2015-10-08

**Authors:** Christoph A. Thaiss, Maayan Levy, Eran Elinav

**Affiliations:** Immunology Department, Weizmann Institute of Science, Rehovot, Israel; Geisel School of Medicine at Dartmouth, UNITED STATES

## How Do Mammals Organize Their Biological Clocks?

The rotation of the Earth around its own axis creates a fundamental challenge for life on this planet, related to the need of all living organisms to modify their physiology in accordance with daily variations in multiple geophysical properties, including light, temperature, and availability of nutrients. As a consequence, all domains of life have developed intrinsic timing systems (“biological clocks”) in order to efficiently adapt organismal activity to the fluctuating environmental conditions [1]. The mechanisms by which different organisms have solved this challenge are manifold. In mammals, the molecular clock is classically understood as a network of transcription factors that are rhythmically operational in virtually all cells of the body [2]. Specifically, the core circadian clock is constituted by the transcription factors Period/Cryptochrome and BMAL1/CLOCK, which not only control each other’s circadian expression pattern but also drive rhythmic oscillations in a large number of target genes, thereby orchestrating the daily activity profile of a cell [3]. Interestingly, mammalian molecular clocks are autonomous and self-sustained but can be entrained by external stimuli, such as light and nutrient availability, in order to adapt organism-intrinsic rhythmicity to fluctuating environmental conditions.

## Are There Biological Clocks in Bacteria?

Until about 25 years ago, evidence for a biological clock had only been found in eukaryotes. Since then, however, bacterial circadian clocks have been well documented. Similar to eukaryotic clocks, they are endogenous, self-sustained, and can be entrained by environmental conditions. Of note, bacterial circadian rhythms have so far primarily been described in organisms that are light responsive, namely the cyanobacterium *Synechococcus elongates* [4]. In this bacterium, the molecular clock consists of three proteins (KaiA, KaiB, and KaiC). Remarkably, their oscillatory activity can be sustained in the absence of transcription and is characterized by rhythmic phosphorylation patterns [5]. In vitro reconstitution of this three-member clock leads to sustained rhythmic activity [6]. Of note, this rhythmic phosphorylation adapts to availability and intracellular storage of nutrients [7]. Although potential homologs in the genomic sequence of other bacteria have been found, their characterization awaits further studies. In addition, oxidation-reduction cycles of peroxiredoxin proteins have been found to be circadian across all domains of life [8]. Thus, circadian systems have putatively evolved in multiple distinct ways in different forms of life.

## What Happens to Biological Clocks in Places Where Eukaryotes and Prokaryotes Share Their Habitats?

**Fig 1:**
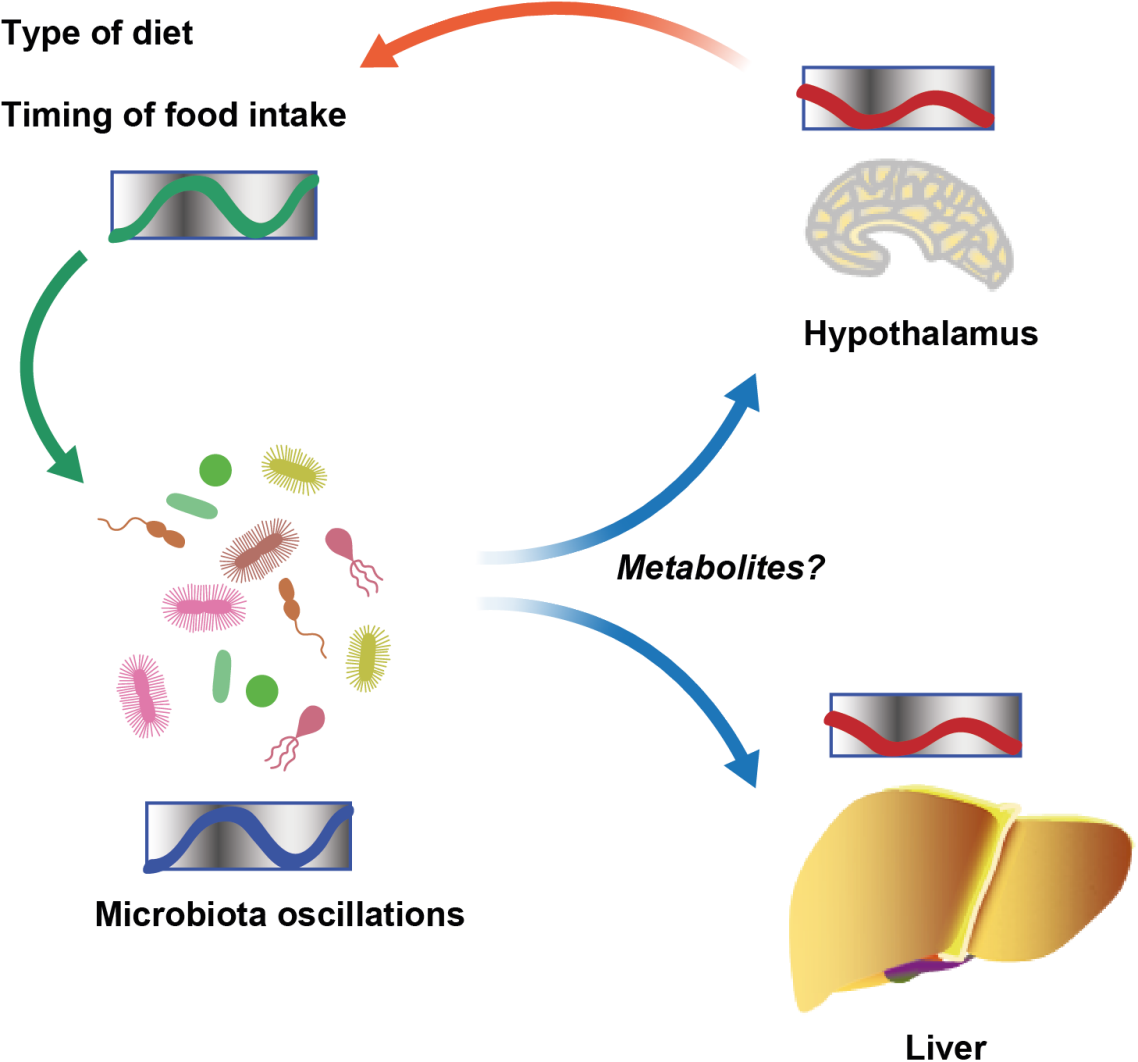
Schematic showing diurnal cross talk between host and intestinal microbiota.

The study of diverse molecular clocks across the domains of life is especially interesting in scenarios in which prokaryotes and eukaryotes interact to form symbiotic communities. The first insight into such cross talk involving clock systems regulated across organisms came from a study of the squid *Euprymna scolopes* and its luminous endosymbiont *Vibrio fischeri* [9]. *E*. *scolopes* encodes genes for cryptochromes, whose oscillatory expression pattern is synchronized with and depends on symbiont luminescence in the light organ of the squid. Further evidence came from studies of the intestinal microbiota in mice, where it was observed that gut microbial colonization influences rhythmic signaling events in the ileal epithelium downstream of toll-like receptors (TLRs). This, in turn, regulates the organization of molecular clock activity and glucocorticoid production in the intestine [10]. The microbiota also impacts clock gene expression beyond the gastrointestinal track. Germ-free mice, which are born and raised under strictly sterile conditions in the absence of microorganisms, feature alterations in clock gene expression in the liver and the hypothalamus [11]. Together, these studies suggest that microbial colonization has a previously unappreciated function in the maintenance of circadian rhythms in the eukaryotic hosts. The underlying mechanisms for this activity remain obscure (Fig 1).

Recently, we uncovered that the circadian interactions between host and microbiota are not solely restricted to microbial control of host clock function but rather constitute a bidirectional cross talk [12]. We and others demonstrated that the intestinal microbiota undergoes rhythmic fluctuations in taxonomic composition and, consequently, genomic coding capacity [11–13]. As a result, different times of the day feature distinct microbial community configurations and microbiota functions. Thus, in addition to the intrinsic circadian timing systems identified in cyanobacteria, the intestinal microbiota undergoes community-scale rhythmicity on the level of metagenome composition [14]. These compositional fluctuations are controlled by the circadian clock of the host, since they cease in the absence of a functional host molecular clock [12]. Furthermore, the type of diet and the rhythmicity of food intake are major drivers of the oscillations in the intestinal microbial ecosystem [12,13]. In addition, recent insights using parenteral feeding raise the possibility that further, still-unidentified host factors are involved in regulating microbial oscillations [11]. Taken together, symbiotic microbial colonization is required for circadian homeostasis of the host. In turn, a functional host circadian clock ensures periodic oscillations of its symbiotic microbial ecosystem (Fig 1).

## How Does the Biological Clock Influence the Antimicrobial Response?

While the above examples represent homeostatic diurnal cross talk between eukaryotes and prokaryotes in symbiotic communities, it is interesting to consider whether the underlying regulatory principles also apply to the specific cases in which the ecosystem is invaded by pathogens. Such situations are characterized by loss of mucosal homeostasis and the instigation of a rapid immune response. Indeed, many aspects of antimicrobial pathway and innate immune response regulation involve the circadian clock [15,16], as was first discovered in *Drosophila* [17]. In mice, the molecular clock regulates diurnal expression of proinflammatory genes in macrophages, thereby generating characteristic profiles of cytokine expression over the course of a day [18]. Furthermore, leukocyte abundance in the circulation and recruitment to peripheral tissues underlies strong circadian fluctuation [19]. The circadian clock also controls the expression of innate immune receptors, as has been described for TLR9 [20]. As a result, inflammatory responses, including susceptibility to sepsis, are strongly influenced by the time of day [19,20]. Major insights came from the study of cell-intrinsic clock functions in immune cells. Inflammatory monocytes with a cell type-specific deletion of *Bmal1* feature a disrupted diurnal trafficking pattern, predisposing mice to inflammatory diseases [21].

In addition to the circadian regulation of innate immunity, the development of adaptive immune cells, in particular T helper (T_H_) 17 cells, has also been suggested to be regulated by the circadian clock. The clock transcription factor REV-ERBα controls the T_H_17-controlling transcription factor RORγt through NFIL3 [22], such that T_H_17 lineage specification is under circadian control. Recently, however, it has been found that mice with a T cell-specific deletion of *Bmal1* do not feature defective adaptive immune responses [23], raising the possibility that the observed regulation of T_H_17 cells may involve cell-extrinsic factors.

As a result of the circadian variation in immune system potency, the susceptibility of the host to pathogenic infection varies over the course of a day. For instance, the degree of the immune response to oral infection with *Salmonella* Typhimurium is more pronounced when the infection occurs during the active phase of the host [24], likely to anticipate a higher risk to acquire foreign microbial elements during the time of food intake. The time of day therefore also affects the ability of the host to clear infection. For both *S*. Typhimurium and *Listeria monocytogenes*, it has been found that pathogen clearance varies with circadian time, a phenomenon that is not apparent in mice with genetic deletion of clock components [21,24]. Whether the immune system is also involved in circadian variations in the antimicrobial response against commensals and whether such responses may regulate diurnal rhythms of the microbiome remain elusive.

## What Are the Consequences of Interkingdom Diurnal Rhythmicity?

The findings described above establish diurnal activity as a new principle in host–microbial interactions. The microbiota has emerged as a major mediator in the interaction of the host with its environment, so rhythmic adaptation of the eukaryotic and prokaryotic part of the “metaorganism”to the time of day might have been an important selective feature during evolution. There are three important conclusions that can be drawn from the first studies in this emerging field. First, it seems that biological clock systems are at work in a large range of biological contexts, ranging from cell-intrinsic clocks consisting of only three proteins, to larger transcriptional networks, oscillations at the level of organ function, and whole-organism behavior, to even symbiotic community-wide cross regulation of diurnal activity between different domains of life. The full spectrum of rhythmicity levels that are featured by the microbiome remains to be investigated and might likewise encompass important cell-intrinsic, transcriptomic, and metabolomics oscillations. Such insights will be fundamental for the mechanistic understanding of daily rhythmicity and its consequences in host–microbial interactions.

Second, the diurnality in host–microbiota interactions might, at least in part, account for the critical role that the biological clock plays in regulation of the host’s metabolic homeostasis. Indeed, microbiota diurnal rhythms have been recently suggested to exist in humans, to be disturbed upon clock disruption of the host, and consequently to drive metabolic aberrations [12]. Thus, in addition to morbidity induced by pathogens, microbiome diurnal rhythmicity may also influence noninfectious disease pathogenesis, including common multifactorial diseases that have been linked to disruptions in the circadian clock [14].

Finally, the findings of interdependent diurnal behavior in symbiotic communities may have another important consequence. They suggest that a stable state in such an ecosystem is not characterized by the static maintenance of community structure and function, but rather by hour-scale fluctuations around a homeostatic set point. Such an oscillating system might be more capable of meeting the constraints imposed on the community by environmental and geophysical variations over the course of a day, since deviations from the normal state might be more rapidly achieved by modifying the amplitude or frequency of rhythmically occurring oscillations.

The field of circadian rhythms in host–microbial interactions is still in its infancy, and the major functional principles and mechanisms have yet to be understood. Nonetheless, the first studies have already shown the great promise that this field holds for our understanding of the factors regulating host–microbial interactions, ecosystem stability, susceptibility to infection, and metabolic disorders—a list that may be further expanded in the years to come.
